# Factors associated with the uptake of COVID-19 vaccination, testing and medical care among Myanmar migrants in Japan: a cross-sectional study

**DOI:** 10.1186/s41182-024-00621-4

**Published:** 2024-08-06

**Authors:** Moe Moe Thandar, Azusa Iwamoto, Haru Angelique Hoshino, Kyoko Sudo, Mihoko Fujii, Miwa Kanda, Saki Ikeda, Masami Fujita

**Affiliations:** 1 Bureau of International Health Cooperation, National Center for Global Health and Medicine, Tokyo, Japan; 2 Center for Field Epidemic Intelligence, Research and Professional Development, National Institute of Infectious Diseases, Tokyo, Japan; 3 Migrants’ Neighbor Network & Action (MINNA), Tokyo, Japan

**Keywords:** COVID-19, Test, Care, Vaccination, Migrants, Myanmar, Japan

## Abstract

**Background:**

Migrants are individuals who move to a different country from their usual place of residence. Japan’s migrant population, particularly technical interns and students, has been growing. Even before the pandemic, previous studies have highlighted difficulties faced by migrants in utilizing healthcare services. This study examined the uptake of COVID-19 vaccination, testing, and medical care among Myanmar migrants in Japan; identified the factors associated with this uptake; and described the difficulties encountered when utilizing these services.

**Methods:**

A cross-sectional study was conducted between March and April 2023 targeting Myanmar migrants over 18 years residing in Japan for more than 6 months. An online self-administered questionnaire in Myanmar language covered socioeconomic characteristics; history of COVID-19 vaccination, testing, and medical care; and difficulties encountered while utilizing these services with multiple-choice questions. Multivariate logistic regression analysis was performed separately to identify the factors associated with the uptake of COVID-19 vaccination, testing, and medical care.

**Results:**

Among the 207 participants, 52% (*n* = 108) were under 30 years, 30% (*n* = 62) were male, and 31% (*n* = 65) were low-skilled workers (technical interns and students). Overall, 91% (*n* = 189) had received the COVID-19 vaccination, 76% (*n* = 157) had been tested for COVID-19, and 43% (*n* = 68) tested positive. However, only 77% (*n* = 52) of COVID-19 patients sought medical care. Participants under 30 years of age were less likely to receive the COVID-19 vaccine compared to those aged 30 and older (adjusted odds ratio [aOR] 0.10, 95% confidence interval [CI] 0.01–0.88, *p* = 0.038). Low-skilled workers were less likely to seek medical care compared to those holding other categories of residential status (aOR 0.12, 95% CI 0.02–0.79, *p* = 0.027). Among service users, 5% faced difficulties with COVID-19 vaccination, 10% with testing, and 17% with receiving medical care. Long waiting times and complex reservation processes were the main difficulties encountered.

**Conclusions:**

The uptake of COVID-19 vaccination, testing, and medical care in this sample was reasonably good. However, individuals under 30 years of age showed lower uptake of vaccination, while low-skilled workers had lower uptake of medical care. Strengthening education and support for young migrants and low-skilled workers regarding COVID-19 and other infectious diseases is essential, especially in workplaces and educational institutions.

**Supplementary Information:**

The online version contains supplementary material available at 10.1186/s41182-024-00621-4.

## Background

The number of migrants in Japan has been increasing in recent decades [1]. As of December 2022, there were approximately 3 million migrants in Japan, of which 1.8 million were migrant workers [2]. Migrants in this study referred to “international migrants” as defined by the United Nations Department of Economic and Social Affairs “any person who changes his or her country of usual residence” excluding movements that are due to “recreation, holiday, visits to friends and relatives, business, medical treatment or religious pilgrimages” [3]. Migrant workers encompassed a diverse group of migrants, including those working in professional or technical fields, specified skilled workers, technical interns, and international students or migrants working part-time jobs [4]. Technical interns and international students at Japanese language preparatory schools are a large proportion of migrant workers as a result of a series of policy packages, such as the “Technical Intern Training Program” and the “300,000 International Student Policy”, which were launched in 1993 and 2008, respectively [4–6]. Although technical interns and students are generally young and healthy [7], they should be regarded as vulnerable mainly because they have low-income jobs, are low-skilled, often have poor working conditions, live in crowded accommodations, and are vulnerable to the spread of infectious diseases [8, 9].

Studies conducted early in the COVID-19 pandemic suggest that migrants have limited access to COVID-19 related healthcare compared with the general population of the host country [10, 11]. Migrants in Japan, including technical interns and students, have faced challenges in accessing healthcare services even before the pandemic, particularly due to language or health insurance-related barriers [8, 12–14]. Japan’s basic policy on COVID-19 involves containing the spread of the infection, sustaining the healthcare system, and prioritizing care for severely ill patients [15]. Vaccination services for COVID-19 are provided free of charge to individuals receiving vaccine vouchers [16]. The costs associated with COVID-19 testing and medical care are basically covered for patients whose tests were positive [17]. However, the guidelines for testing and medical care have been adjusted throughout the pandemic. For instance, initially, polymerase chain reaction (PCR) tests were offered free of charge only to symptomatic individuals or asymptomatic individuals with close contacts of confirmed cases. Subsequently, in 2021, free PCR testing for asymptomatic individuals was implemented nationwide in Japan following the surge of Omicron variant [18, 19].

Vaccination and testing play crucial roles in mitigating and preventing the transmission and outbreak of COVID-19 [20–22]. Understanding the uptake of these services can contribute to the design and implementation of policies and programmes to increase coverage, address context-specific drivers and barriers, evaluate the effectiveness of strategies, and advocate for and mobilize resources [23]. Socioeconomic characteristics, including age, education, occupation, and income, can influence the uptake of COVID-19 vaccination and testing [20, 24–28]. Difficulties may arise when utilizing these services, such as transportation difficulties, language difficulties, financial difficulties, and the inability to book an appointment for a vaccine or test [29, 30]. Data on the uptake of COVID-19 vaccination, testing, and subsequent medical care for COVID-19 positive patients among migrants in Japan remain limited [31] and the factors associated with the uptake of these services and the difficulties they face when utilizing these services are not well documented.

As of December 2022, the population of Myanmar migrants in Japan was recorded at 56,239 individuals [32]. In the same year, Myanmar ranked among the top ten countries contributing migrant workers in Japan, with 47,498 Myanmar workers representing approximately 2.6% of total migrant workforce [33]. Furthermore, Myanmar was one of the three main countries with the highest year-on-year increase rates [33]. However, very few studies have been conducted among Myanmar migrants in Japan [34, 35]. This study aimed to examine the uptake of COVID-19 vaccination, testing, and medical care; identify the factors associated with the uptake; and describe the perceived difficulties in utilizing these services among Myanmar migrants in Japan.

## Methods

### Study design and participants

We conducted a cross-sectional online survey among Myanmar migrants in Japan from 1 March to 30 April 2023. Myanmar migrants aged 18 years or older who had been living in Japan for more than 6 months were eligible to participate in the survey. The 6-month duration was selected to allow the participants sufficient time to experience the Japanese medical system including vaccination.

### Sampling

The snowball and convenience sampling methods were employed to enrol participants from all prefectures in Japan. For snowball sampling, online survey information was circulated through Facebook by several voluntary associations in Japan (Myanmar Youth and Student Association, Myanmar Parents and Children Community, Japan Myanmar Help Desk, and Priman in Japan) that offer a wide range of social welfare support for Myanmar migrants in Japan. Those who completed the survey invited their friends and colleagues to participate in the study. For convenience sampling, a research assistant handed out printed leaflets to visitors and customers visiting a Myanmar festival and two restaurants in Tokyo. In both sampling methods, participants accessed the online survey through a link or QR code that led to a webpage containing an explanatory document, an electronic consent form, and a self-administered questionnaire in the Myanmar language. Participation was entirely voluntary, and no incentives were given to the respondents.

### Data collection tool

The researchers first developed a questionnaire in Japanese, referring to previous studies [24, 26, 36]. The Japanese questionnaire was translated into Myanmar using a professional translation service. Microsoft Forms, which provides online questionnaire design and survey functions, was used. To verify the readability and length of the questionnaire, an online pilot-test was conducted with five Myanmar residents in Japan. The feedback obtained from these participants was used to revise the questionnaire before it was distributed for the online survey. These participants were not included in the final analysis. It took approximately 10 min to complete the survey. The questionnaire included 46 questions regarding respondents’ socioeconomic characteristics, underlying diseases, health insurance, unmet healthcare needs, uptake of COVID-19 vaccination, testing, and medical care, reasons for not utilizing these services, and difficulties when utilizing these services. Socioeconomic characteristics included age, sex, education, occupation, residential status, length of stay in Japan, self-rated Japanese proficiency, and monthly household income. The assessment of unmet healthcare needs was evaluated using a binary question (Yes or No): “In the past 12 months, have you encountered a situation where you desired but were unable to consult a doctor in Japan for a health-related issue?” [13, 37, 38]. Individuals who responded, “Yes”, were defined as having encountered unmet healthcare needs, and subsequent inquiries were made to clarify the reasons behind with multiple-choice question. Uptake of COVID-19 vaccination was assessed using a binary question (Yes or No): “Have you ever been vaccinated against COVID-19 in Japan?” Uptake of COVID-19 testing was assessed using a binary question (Yes or No): “Have you ever received a COVID-19 test (PCR or antigen test, including self-examination kit) in Japan?” Uptake of COVID-19 medical care was assessed using two steps binary questions (Yes or No): “Have you ever been tested positive for COVID-19 in Japan?” and “If you answered “Yes” in the previous question, did you seek medical care?” The respondents were asked multiple-choice questions about their reasons for not utilizing these services. In instances where respondents had utilized these services, we inquired about the difficulties they experienced with multiple-choice questions: “What difficulties did you have in using this service in Japan?”. All the multiple-choice questions in this survey were designed with open-ended response fields to capture details of the respondent’s reasons or difficulties that were not encapsulated within the provided options. We did not collect any information that could identify the participants.

### Statistical analysis

All statistical analyses were performed using Stata SE version 18 [39]. We used descriptive statistics to show the characteristics of the participants, uptake of COVID-19 vaccination, testing, and medical care, reasons for not receiving these services, and perceived difficulties in utilizing these services using frequencies and proportions for each variable. The residential status of participants was categorized into five groups: (1) low-skilled workers (technical interns and students in language school, college, or university); (2) high-skilled workers (specialist in technical or humanities or international services, specific skilled worker, professor, researcher, and official); (3) family or status-based (Japanese, spouse or child of Japanese national, permanent resident, spouse or child of permanent resident, and dependent); (4) undocumented migrants (temporary release and no visa); and (5) others (who did not mention their residential status). For further analysis, variables were recategorized as binary variables.

Chi-square or Fisher’s exact tests were used to describe the differences in the uptake of COVID-19 vaccination, testing, and medical care based on socioeconomic characteristics. We then performed separate bivariate regression models to identify the potential determinants of the uptake of each service. Variables attaining significance (*p* < 0.25 in the bivariate regression models) were included in the multivariable models. Variables were retained in the final model based on statistical significance and collinearity among the potential determinants (variables with a variance inflation factor > 2.5 were dropped). Finally, multivariate logistic regression models were fitted to investigate the determinants of uptake of COVID-19 vaccination, testing, and medical care separately. Goodness of fit was assessed using the Hosmer–Lemeshow test. The estimates of the determinants of uptake of COVID-19 vaccination, testing, and medical care were summarized using adjusted odds ratios (aOR) and their 95% confidence intervals (CI). Two-tailed analyses were used to calculate *p*-values, with *p* < 0.05 considered statistically significant.

## Results

### Participant characteristics

A total of 211 people accessed the online survey link; one respondent did not provide informed consent and three respondents did not meet the inclusion criteria. A total of 207 participants living in 27 prefectures of Japan were included in the analysis. Of these, 88% (*n* = 182) lived in the Kanto area (the Tokyo metropolitan area and its surrounding prefectures), and 63% (*n* = 131) were recruited through Facebook (Table 1). More than half of the participants (52%, *n* = 108) were under 30 years of age, and 30% (*n* = 62) were male. Regarding residential status, 47% (*n* = 98) were high-skilled workers, 31% (*n* = 65) were low-skilled workers, 18% (*n* = 38) had a family or status-based residential status, and 2% (*n* = 4) did not mention their residential status. There were only two undocumented migrants (1%) who were temporarily released or did not have any visas. Forty-six per cent (*n* = 96) of the participants had a median length of stay in Japan of 4–9 years and 63% (*n* = 131) had intermediate-level Japanese language proficiency.

**Table 1:** Socioeconomic characteristics of participants (*n* = 207)

	*n*	%
Recruitment		
Facebook	131	63.3
Printed leaflets	76	36.7
Location of residence		
Kanto area*	182	87.9
Others	25	12.1
Age (years)		
Under 30	108	52.1
30–39	66	31.9
40 and above	33	15.9
Sex		
Female and unidentified	145	70.0
Male	62	30.0
Highest education		
High school	36	17.4
College or university	140	67.6
Master’s degree and above	31	15.0
Residential status		
Low-skilled workers	65	31.4
High skilled workers	98	47.4
Family or status-based	38	18.4
Undocumented migrants	2	0.9
Others	4	1.9
Length of stay in Japan		
6 months to 3 years	70	33.8
4–9 years	96	46.4
10 years and above	41	19.8
Japanese language proficiency (self-rated)		
Not at all (No)	7	3.4
Not well (Limited)	33	15.9
Well (Intermediate)	131	63.3
Very well (Native)	36	17.4
Working status		
Not working	17	8.2
Employed full time	132	63.3
Employed part time	50	24.2
Self-employed/freelance	8	4.4
Income per month (Yen)		
Less than 150,000	47	24.7
150,000–250,000	53	27.9
More than 250,000	48	25.3
Don’t want to answer	42	22.1
Living situation		
Alone	89	43.0
With family	54	26.1
With friends/colleague/employer	64	30.9

^*^The Kanto area encompasses the Tokyo metropolitan area and its surrounding prefectures, namely Ibaraki, Tochigi, Gunma, Saitama, Chiba, and Kanagawa

### Underlying diseases, health insurance, and unmet healthcare needs

Less than 5% (*n* = 9) reported underlying diseases such as chronic lung diseases, cardiovascular diseases, and mental health conditions (Table 2). The majority of the participants (98%, *n* = 203) were covered by Japanese health insurance, of whom 20% (*n* = 40) reported difficulty in paying their health insurance premiums. Approximately 88% (*n* = 182) of the total participants perceived that they could receive healthcare in Japan if needed, but there were also unmet healthcare needs in the past 12 months (5%, *n* = 10). Language barriers were the most common reason for unmet healthcare needs (Table S1).

**Table 2:** Underlying diseases, health insurance, and unmet healthcare needs (*n* = 207)

	*n*	%
Underlying diseases*		
No	198	95.7
Yes*	9	4.3
Health insurance in Japan		
No	4	1.9
Yes	203	98.1
Financial difficulty for health insurance		
No	163	80.3
Yes	40	19.7
Perceived accessibility to health care		
No	25	12.1
Yes	182	87.9
Unmet healthcare need in the past 12 months		
No	197	95.2
Yes	10	4.8

^*^Multiple selection: chronic lung diseases (2), cardiovascular diseases (2), mental health conditions (2), unspecified (2), chronic liver disease (1), diabetes (1) and obesity (1)

### Uptake of COVID-19 vaccination, testing, and medical care

**Fig. 1:**
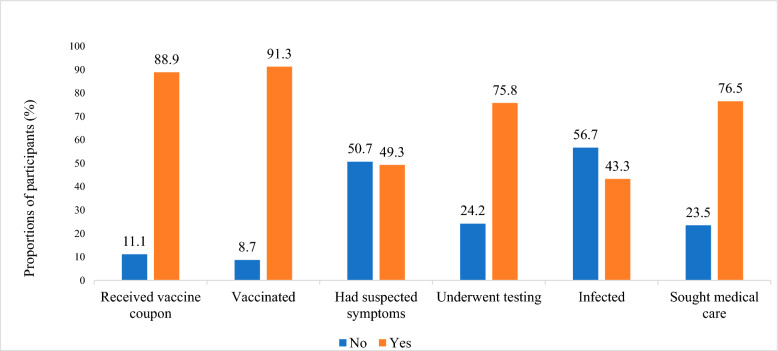
Proportions of participants who were vaccinated, underwent testing and sought medical care for COVID-19

Overall, 89% (*n* = 184) of the participants reported that they had received vaccine coupons and 91% (*n* = 189) reported having received a COVID-19 vaccine in Japan at least once (Fig. 1). The most common reasons for not receiving the COVID-19 vaccine were lack of knowledge about vaccination centres in Japan and having already received the recommended vaccine doses in their home country (Table S1). Approximately 49% (*n* = 102) of the participants had COVID-19 suspected symptoms and 76% (*n* = 157) had been tested for COVID-19. Of the tested patients, 68 (43%) were positive, but only 52 (77%) sought medical care. The most common reason for not receiving test or seeking medical care was that they could not be bothered. Differences in the uptake of COVID-19 vaccinations, testing, and medical care according to socioeconomic characteristics are described in Table 3. The Chi-square analysis revealed a statistically significant lower uptake of COVID-19 vaccination among specific demographic groups: individuals younger than 30 years, low-skilled workers (technical interns and students), those with a residency duration in Japan of less than 3 years, those with low Japanese language proficiency, and non-full-time workers. Additionally, the uptake of COVID-19 testing was significantly lower among males, individuals with a residency duration in Japan of less than 3 years, and those with low Japanese language proficiency. Moreover, the uptake of COVID-19 medical care was significantly lower among low-skilled workers and those with monthly incomes of less than 250,000 yen.

**Table 3:** Differences in the uptake of COVID-19 related health care services according to socioeconomic characteristics

	Uptake of COVID-19 vaccination (n = 207)			Uptake of COVID-19 testing (n = 207)			Uptake of COVID-19 medical care (n = 68)		
	No (n, %)	Yes (n, %)	p-value	No (n, %)	Yes (n, %)	p-value	No (n, %)	Yes (n,%)	p-value
Location of residence									
Others	4 (16.00)	21 (84.00)	0.244	6 (24.00)	19 (76.00)	0.985	3 (42.86)	4 (57.14)	0.342
Kanto area	14 (7.69)	168 (92.31)		44 (24.18)	138 (75.82)		13 (21.31)	48 (78.69)	
Age									
30 years and older	2 (2.02)	97 (97.98)	0.001	23 (23.23)	76 (76.77)	0.767	7 (19.44)	29 (80.56)	0.400
Under 30 years	16 (14.81)	92 (85.19)		27 (25.00)	81 (75.00)		9 (28.13)	23 (71.88)	
Sex									
Female and unidentified	11 (7.59)	134 (92.41)	0.386	29 (20.00)	116 (80.00)	0.033	13 (26.00)	37 (74.00)	0.529
Male	7 (11.29)	55 (88.71)		21 (33.87)	41 (66.13)		3 (16.67)	15 (83.33)	
Highest education									
High school	4 (11.11)	32 (88.89)	0.525	7 (19.44)	29 (80.56)	0.468	1 (10.00)	9 (90.00)	0.432
College/university and above	14 (8.19)	157 (91.81)		43 (25.15)	128 (74.85)		15 (25.86)	43 (74.14)	
Residential status									
Others	5 (3.52)	137 (96.48)	0.001	32 (22.54)	110 (77.46)	0.421	9 (16.98)	44 (83.02)	0.017
Low-skilled workers	13 (20.00)	52 (80.00)		18 (27.69)	47 (72.31)		7 (46.67)	8 (53.33)	
Length of stay in Japan									
6 months to 3 years	14 (20.00)	56 (80.00)	< 0.001	24 (34.29)	46 (65.71)	0.015	5 (45.45)	6 (54.55)	0.061
4 years and above	4 (2.92)	133 (97.08)		26 (18.98)	111 (81.02)		11 (19.30)	46 (80.70)	
Japanese language proficiency									
Not at all or not very well	7 (17.50)	33 (82.50)	0.028	17 (42.50)	23 (57.50)	0.003	2 (25.00)	6 (75.00)	0.917
Well or very well	11 (6.59)	156 (93.41)		33 (19.76)	134 (80.24)		14 (23.33)	46 (76.67)	
Type of work*									
Part-time/self-employed/freelance	9 (15.52)	49 (84.48)	0.010	19 (32.76)	39 (67.24)	0.068	2 (11.76)	15 (88.24)	0.485
Full time	6 (4.55)	126 (95.45)		27 (20.45)	105 (79.55)		11 (24.44)	34 (75.56)	
Income**									
Less than 250,000	12 (12.00)	88 (88.00)	0.061	22 (22.00)	78 (78.00)	0.685	8 (26.67)	22 (73.33)	0.016
More than 250,000	1 (2.08)	47 (97.92)		12 (25.00)	36 (75.00)		0	19 (100)	
Living situation									
Alone	11 (12.36)	78 (87.64)	0.104	24 (26.97)	65 (73.03)	0.412	9 (31.03)	20 (68.97)	0.208
With family/friend/college/employer	7 (5.93)	111 (94.07)		26 (22.03)	92 (77.97)		7 (17.95)	32 (82.05)	

^*^Excluding “not working”

^**^Excluding “don’t want to answer”

### Factors associated with the uptake of COVID-19 vaccination, testing, and medical care

In multivariable logistic regression, individuals under 30 years of age were less likely to receive COVID-19 vaccination compared to those aged 30 and older (aOR 0.10, 95% CI 0.01–0.88, *p* = 0.038) and low-skilled workers were less likely to seek COVID-19 medical care compared to those holding other categories of residential status (aOR 0.12, 95% CI 0.02–0.79, *p* = 0.027) (Table 4). However, no factors were associated with COVID-19 testing in the adjusted regression model.

**Table 4:** Factors associated with the uptake of COVID-19 vaccination, testing, and medical care

	Uptake of COVID-19 vaccination (*n* = 207)			Uptake of COVID-19 testing (*n* = 207)			Uptake of COVID-19 medical care (*n* = 68)		
	aOR	95% CI	*p*-value	aOR	95% CI	*p*-value	aOR	95% CI	*p*-value
Age									
30 years and older	Reference								
Under 30 years	0.10	0.01–0.88	0.038	0.91	0.40–2.03	0.810	1.04	0.21–5.16	0.963
Sex									
Female and unidentified	Reference								
Male	0.61	0.16–2.37	0.475	0.62	0.29–1.32	0.216	8.86	0.47–168.1	0.146
Residential status									
Others	Reference								
Low-skilled workers	0.38	0.09–1.68	0.205	0.99	0.38–2.57	0.985	0.12	0.02–0.79	0.027
Japanese language proficiency									
Not at all or not very well	Reference								
Well or very well	1.41	0.38–5.29	0.602	2.19	0.92–5.24	0.077	2.75	0.17–44.69	0.477
Type of work*									
Part-time/self-employed/freelance	Reference								
Full time	2.81	0.82–9.65	0.101	1.57	0.73–3.37	0.248	0.32	0.04–2.35	0.261

aOR: adjusted odds ratio

^*^Excluding "not working"

### Difficulties encountered when utilizing COVID-19 vaccination, testing and medical care services

It was observed that 5% of individuals who received vaccination, 10% of those who underwent testing, and 17% of those who sought medical care encountered difficulties, with the most common reasons being the long waiting time to receive these services and difficulties with the appointment process (Table 5). Among the 68 participants with COVID-19, 21% encountered difficulties during hospitalization, hotel, or home isolation. The most common reason was unavailability of food, followed by unavailability of quarantine facilities. Moreover, 18% had difficulties after returning to their daily lives, with the most common reason being post-COVID-19 conditions.

**Table 5:** Difficulties encountered when utilizing g COVID-19 vaccination, testing and medical care services

	Number of participants	Number of participants reported difficulties	Difficulties (multiple answer choices)
Among those who were vaccinated	189	*n* = 9 (5%)	Difficulties in booking process [6]
			Long waiting time for vaccination [4]
			Language barrier [1]
Among those who were tested	157	*N* = 16 (10%)	Difficulties in booking process [4]
			Cost of testing [4]
			Administrative requirements to check document related to visa [2]
			Language barrier [1]
Among those who seek medical care	52	*n* = 9 (17%)	Long waiting time for receiving medical care [7]
			Cost of medical care [2]
			Language barrier [1]
			Ohers [1]
During hospitalization or home or hotel isolation	68	*n* = 14 (21%)	Meals [10]
			Can't find a quarantine place [9]
			Not sure what to do next [4]
			Difficult to contact health centre [1]
After returning to daily life after infection	68	*n* = 12 (18%)	Post-COVID-19 symptoms [9]
			Income [2]
			Living [2]
			Work [1]
			Psychological problems [1]
			Being talked about, discriminated against [1]

## Discussion

This is the first study that explored the uptake of COVID-19 related healthcare services among the Myanmar migrants in Japan. Among the participants of this study, the coverage of COVID-19 vaccination, testing, and medical care exceeded 75%. However, the uptake of vaccination was lower in the younger population (under 30 years of age), and the uptake of medical care was lower in low-skilled workers (technical interns and students). This study also highlights health system-related difficulties in utilizing these services.

In early 2023, 2 years after the rollout of COVID-19 vaccination in Japan, 91% of the participants in this study had received at least one dose of the COVID-19 vaccine in Japan. However, those under 30 years of age were less likely to receive the COVID-19 vaccination than their counterparts aged 30–69 years. Previous studies also reported the differences in COVID-19 vaccine uptake between older and younger migrant population, however, the underlying reasons contributing to these findings were not explored [24, 25, 40]. In this study, most of the participants under 30 years of age were technical interns or students who had recently moved to Japan. The main reasons for not receiving the COVID-19 vaccine in Japan were a lack of knowledge about the vaccine centres in Japan and having received the recommended vaccine doses in their home country. Unfamiliarity with the Japanese healthcare system is one of the barriers to healthcare uptake by migrants [41]. Furthermore, another study highlighted insufficient awareness among young migrants in Japan regarding the locations of mass vaccination sites [42]. Therefore, potential solutions for newcomers are to provide information in multiple languages or in “Yasashi-i Nihongo”, which means simplified Japanese [12], through their workplaces and schools, and to increase interpretation services and support through social workers [43].

In this study, one in four Myanmar migrants diagnosed with COVID-19 did not seek medical care. Low-skilled workers, including technical interns and students, are less likely to seek medical care. The most common reason reported in this study was that the participants considered the procedure unnecessary to do so. Some typical barriers to migrants, such as language barriers, financial constraints, and a lack of knowledge regarding where to obtain medical care [24], were less commonly reported by the participants. This indicates that their perception of COVID-19 was not aligned with its severity despite the infection being considered fatal. This finding highlights the need to improve education on COVID-19 and other infectious diseases.

The difficulties encountered by the study participants when utilizing COVID-19 vaccination, testing, and medical care services were identified in this study. Health system-related difficulties, particularly long waiting times and complex reservation processes, are most often mentioned. Similar difficulties have been reported among Nepalese migrants in Japan [41]. For example, in the context of COVID-19 vaccination, the reservation process involved the issuance of vaccine vouchers by municipality offices, recognition of received vaccine vouchers, online reservation of vaccination venues base on availability, and completion of pre-vaccination screening questionnaire, with all instructions provided in Japanese [31]. In contrast to health system-related difficulties, difficulties due to administrative requirements, language barrier, and financial constraints were mentioned less frequently by the participants. This may be attributed to Japan’s efforts to provide free vaccination and medical care for COVID-19, and to the socioeconomic characteristics of Myanmar migrants in this study.

It is important to note that the characteristics of the participants in this study differed from the overall distribution of Myanmar migrants in Japan. Specifically, as of December 2022, low-skilled workers constituted the largest group of Myanmar migrants in Japan (41%), followed by high-skilled workers (26%) and those with family or status-based residential status (13%) [32]. However, in our study, the largest group consisted of high-skilled workers (47%), followed by low-skilled workers (31%), and those with family or status-based residential status (18%). Accordingly, it is important to cautiously consider the study findings, acknowledging that over half of the study participants were living in favourable conditions in Japan, with relatively stable income and proficient language abilities.

Given the current political and economic situation in Myanmar, a significant number of people seek employment and educational opportunities in Japan. Additionally, many Myanmar migrants residing in Japan face challenges in returning to their home country. Therefore, further research is needed to understand the health issues, health-related behaviour, and uptake of healthcare services across subgroups of Myanmar migrants in Japan.

The online survey through the Facebook pages of Myanmar voluntary associations did not adequately capture low-skilled workers and undocumented migrants. Although we targeted migrant population with heterogeneous backgrounds nationwide, the snowball sampling on Facebook primarily attracted high-skilled workers. Remarkably, we observed a slight increase in low-skilled worker participation when Japanese language teachers or staff members from the supervising organizations overseeing technical interns circulated our survey information. To engage with students and technical interns, establishing effective communication channels with key persons from Japanese language schools or supervising organizations may serve as a practical solution.

We tried to recruit the migrants from hard-to-reach populations by conducting outreach at Myanmar restaurants. A venue-based sampling approach—where the study is conducted in areas with a potentially high concentration of the target population—would be an appropriate method to engage with them [44]. With sufficient time and resources, we hypothesized that visiting several Myanmar restaurants, grocery stores, and religious venues across Japan would allow us to reach a larger number of populations facing challenging conditions. Given their hidden status and fears of detention, collaborative partnerships with community groups supporting these populations are crucial.

In future studies targeting a heterogeneous migrant population, researchers should employ a variety of sampling methods including non-probability sampling methods (such as venue-based sampling, snowball sampling, and quota sampling) and probability sampling method (such as stratified random sampling and cluster sampling) [45]. Furthermore, in-depth interviews with organizations supporting specific migrant groups, such as undocumented migrants, would provide valuable insights into their challenges and coping strategies. This is particularly important given their hard-to-reach status and lack of readily available sampling frame.

### Limitations

This study has the following limitations. First, the sample size was smaller than expected, even though alternative routes were used to reach Myanmar migrants in Japan. This could have been due to the timing of the survey as people’s interest in COVID-19 waned at the end of the COVID-19 pandemic. Second, our findings may not be generalizable to all Myanmar migrants. Although the sample was diverse, approximately 65% of participants were well-off migrants with relatively stable income and proficient language abilities. We could not reach a sufficient number of the most vulnerable groups of migrants, such as undocumented migrants. A more balanced sample that includes them may provide subtler insights. Third, this study did not evaluate the details of COVID-19 vaccination, testing, or medical care in Japan using a simple online questionnaire. Hence, the reasons they did not utilize these services and the difficulties encountered when utilizing these services may change over the course of the pandemic, depending on the Japanese government policy.

## Conclusions

The uptake of COVID-19 vaccination, testing, and medical care in this sample was reasonably good. However, individuals under 30 years of age showed lower uptake of vaccination, while low-skilled workers had lower uptake of medical care. Several difficulties related to the healthcare system were perceived. Strengthening education and support for young migrants and low-skilled workers regarding COVID-19 and other infectious diseases is essential, especially in workplaces and educational institutions. Even though COVID-19 is no longer considered a threatening health crisis, the findings of this study are relevant when preparing for future public health emergencies.

### Supplementary Information

Supplementary Material 1.

## Data Availability

The dataset relating to the current study is available upon request.

## Abbreviations

aOR: Adjusted odds ratio
CI: Confidence interval
COVID-19: Coronavirus disease 2019
PCR: Polymerase chain reaction
