# Supplementary material for: Factors associated with the uptake of COVID-19 vaccination, testing and medical care among Myanmar migrants in Japan: a cross-sectional study
Source: Trop Med Health. 2024 Aug 6;52:53. doi: 10.1186/s41182-024-00621-4 (PMC11302098; doi:10.1186/s41182-024-00621-4)
Supplement: Supplementary file 1 — Supplementary Material 1. [file 41182_2024_621_MOESM1_ESM.docx]

**Supplementary information**

**Table S1. Reasons for not receiving health care**

| **Reasons** | **n (%) (multiple answer choices)** |
| --- | --- |
| **Reasons for unmet health care needs (out of 207 participants)** | **n=10 (4.8%)** |
| Language barrier | 3 |
| Do not know where to receive care | 2 |
| Denied by health care workers during the peak of COVID-19 | 2 |
| Do not know how to receive care | 1 |
| Could not make a reservation | 1 |
| Unclear instruction from health center | 1 |
| Insufficient health care workers | 1 |
| **Reasons for not receiving COVID-19 vaccination (out of 207 participants)** | **n=18 (8.7%)** |
| Have already received recommended vaccine doses in home country | 5 |
| Don’t know where to get vaccinated | 5 |
| Concern about side effects | 4 |
| I am hesitant to be vaccinated/I don’t believe in vaccines | 3 |
| Did not receive vaccine voucher | 3 |
| Illness or vaccination contraindication | 1 |
| Language barrier | 1 |
| **Reason for not being tested for COVID-19 (out of 207 participants)** | **n=50 (24.2%)** |
| Could not be bothered | 39 |
| Did not have time to get tested | 5 |
| Confusion and uncertainty regarding testing guidelines and where to go for testing | 3 |
| Perceived as uninfected | 2 |
| Worried about the cost of testing | 1 |
| Did not get a permission from employer | 1 |
| Inability to communicate in Japanese | 1 |
| Inability to get self-examination kit | 1 |
| **Reason for not seeking medical care for COVID-19 (out of 68 infected)** | **n=16 (23.5%)** |
| Could not be bothered | 7 |
| Confusion and uncertainty regarding guidelines for receiving care and where to go | 5 |
| Denied by health care workers during the peak of COVID-19 | 2 |
| Worried about the cost of medical care | 1 |
| Did not get a permission from employer | 1 |
| Inability to communicate in Japanese | 1 |
